# Behavioural health consultants in integrated primary care teams: a model for future care

**DOI:** 10.1186/s12875-016-0485-0

**Published:** 2016-07-29

**Authors:** Hannah Dale, Alyssa Lee

**Affiliations:** 1 NHS Fife, Department of Psychology, Lynebank Hospital, Halbeath Road, Dunfermline, KY11 4UW UK; 2 School of Medicine, Medical and Biological Sciences Building, University of St Andrews, North Haugh, St Andrews, Fife, KY16 9TF UK

**Keywords:** Primary Care, Behavioural Health, Psychology, Collaboration, Integration, Biopsychosocial, Health Inequalities, Prevention, Service Improvement

## Abstract

**Background:**

Significant challenges exist within primary care services in the United Kingdom (UK). These include meeting current demand, financial pressures, an aging population and an increase in multi-morbidity. Psychological services also struggle to meet waiting time targets and to ensure increased access to psychological therapies. Innovative ways of delivering effective primary care and psychological services are needed to improve health outcomes.

**Summary:**

In this article we argue that integrated care models that incorporate behavioural health care are part of the solution, which has seldom been argued in relation to UK primary care. Integrated care involves structural and systemic changes to the delivery of services, including the co-location of multi-disciplinary primary care teams. Evidence from models of integrated primary care in the United States of America (USA) and other higher-income countries suggest that embedding continuity of care and collaborative practice within integrated care teams can be effective in improving health outcomes. The Behavioural Health Consultant (BHC) role is integral to this, working psychologically to support the team to improve collaborative working, and supporting patients to make changes to improve their health across management of long-term conditions, prevention and mental wellbeing. Patients’ needs for higher-intensity interventions to enable changes in behaviour and self-management are, therefore, more fully met within primary care. The role also increases accessibility of psychological services, delivers earlier interventions and reduces stigma, since psychological staff are seen as part of the core primary care service. Although the UK has trialled a range of approaches to integrated care, these fall short of the highest level of integration. A single short pilot of integrated care in the UK showed positive results. Larger pilots with robust evaluation, as well as research trials are required. There are clearly challenges in adopting such an approach, especially for staff who must adapt to working more collaboratively with each other and patients. Strong leadership is needed to assist in this, particularly to support organisations to adopt the shift in values and attitudes towards collaborative working.

**Conclusions:**

Integrated primary care services that embed behavioural health as part of a multi-disciplinary team may be part of the solution to significant modern day health challenges. However, developing this model is unlikely to be straight-forward given current primary care structures and ways of working. The discussion, developed in this article, adds to our understanding of what the BHC role might consist off and how integrated care may be supported by such behavioural health expertise. Further work is needed to develop this model in the UK, and to evaluate its impact on health outcomes and health care utilisation, and test robustly through research trials.

## Background

Primary care services require a new approach to deliver high quality healthcare that meets modern health needs [1–3]. In the UK, we are currently failing to adequately address the increasing demand for primary health care and the current system appears increasingly unsustainable [3–8]. Factors impacting on this increasing pressure include: an aging population; an increase in multi-morbidity; inequalities in access (impacting people in areas of deprivation to the greatest degree); and financial pressures [4, 9, 10]. The increasing number of patients with complex co-morbid physical and mental health conditions (often along with significant social issues) is pertinent, since there is recognition that more complex patients require longer appointments [11, 12]. Poor psychological health and social difficulties compound physical health problems, making self-management of long-term conditions and behavioural change harder and less likely [8]. Societal-level inequalities contribute significantly to problems such as these and are increasing, with consequent negative impacts on individual and community health, such as low-income populations showing greater ill health [13–16].

Naturally, specific challenges for General Practitioner (GP) surgeries will vary across the UK. However, it is commonly felt that primary care in the UK is not sustainable [1, 4–8, 17]. Indicators of the stress that the current system of primary care in the UK is under are not hard to find; for example, the increasing waits that patients’ face in getting an appointment [18, 19]. Furthermore, across the UK, it is also known that due to factors including GP- retirement and problems recruiting GPs, there will be increasing problems in staffing primary care with sufficient GPs in years to come [17, 20]. Accordingly, there needs to be consideration of new models of working within primary care that may meet the modern health care needs of the UK population [21].

Psychological services in the UK are also under evermore pressure, in particular to meet waiting time targets and increase access to services. Even with programs designed to tackle this, for example, Increasing Access to Psychological Therapies (IAPT), problems remain: waiting times, for instance, often remain long (for example only overall 61 % of people are seen within 28 days in English IAPT services) [22, 23]. Attendance rates within referral based services for psychological/behavioural care can also be poor; research typically shows that missed appointments in mental health care in the UK to vary from around 9 to 36 % [24–27]. Further, for example, a study of people in IAPT services showed that 35 % decline treatment or drop out, and only 19 % of those referred receive two or more therapy sessions [28]. Therefore, services (e.g. IAPT) designed to increase accessibility and reach are, at times, struggling to do so, perhaps in part due to the systems of referral, and the challenges in reaching people with mental (and often physical) health problems [29]. Despite this, primary care may be treating up to 25 % of its patients for mental health problems, and may be the sole care providers for a substantial number of these patients [30, 31]. Inequalities also manifest in psychological services, with patients from hard-to-reach groups, such as areas of higher deprivation, men and some ethnic minorities, less likely to attend appointments [32–35]. Innovative ways of delivering behavioural/psychological care are key to achieving better engagement, improved psychological outcomes and better self-management and lifestyle behaviours.

In this debate article, we will argue that given the problems discussed above in primary care and psychological services, there is a need to move towards a more integrated, collaborative and relationship-based model of primary care. As part of that model, there is the urgent need for a role that focuses on psychological and behavioural aspects of health care within primary care. This role has seldom been argued in relation to primary care in the UK, despite up to two-thirds of deaths in under 75s being avoidable through behaviour change; hence the focus of this paper [36, 37]. We aim to propel its development through presenting: a) a discussion about why integrated care models may be required; b) the evidence around collaborative/relationship-based models of primary care; c) an exploration of work to-date in the UK around integrated care, including our own experience of a developing a psychological/behavioural role within primary care; d) a consideration of the possible benefits and challenges of this approach; and, finally e) a discussion of what we feel is needed to ensure such a model can be implemented successfully in primary care. We hope this will stimulate debate and innovation regarding the potential for this model of primary care delivery in the UK.

## Discussion

### Why move towards integrated care models?

Integrated care models in primary care integrate a multi-professional team designed to treat medical, social and psychological/behavioural issues within one easy-to-access primary care team within community settings. Key to this approach, which has developed significantly in the USA, is embedding non-medical professionals with behavioural/psychological expertise as part of the collaborative primary care team. Currently, National Health Service (NHS) organisations across the UK are exploring different ways of delivering primary care to better meet the needs of patients, and future challenges [4, 38]. The 5 Year Forward plan has prompted the piloting of Multispecialty Community Providers to provide integrated care in the community [4]. Integration of health and social care services - including, in most cases, the management of financial budgets and jointly held accountability - should improve communication, coordination and access to services, supporting primary care patients to live well and thrive in their communities [39, 40]. These go some way towards integrating services, however, as described further below, do not represent fully integrated primary care.

Change is also required at the healthcare delivery level. Organisational change to enable the establishment of a fully integrated model of care would likely enhance partnership working between professions and with patients. This is because an integrated primary care team would prioritise interactions with patients that are relationship-based, empathetic and collaborative. Although incredibly challenging to deliver, this form of care results in better outcomes for patients and reduces health service utilisation [41, 42]. This approach may also enable greater prevention, early intervention and reduced hospital visits, improving care and saving costs [43, 44].

In addition to changes in systemic and structural factors that could improve health, evidence suggests that a proportion of patients may require targeted, sometimes high-intensity, psychological interventions to help them make behavioural changes to improve management of long-term conditions, enhance wellbeing, manage mental health problems and to reduce their risk of developing preventable health conditions (e.g. COPD, CVD and diabetes) [45–50]. Training medical and other primary care staff in isolation to deliver self-management support within primary care may not be sufficient for patients to make significant changes. Ongoing support and/or coaching to change staff behaviour in interactions with patients is likely required [51–54]. This suggests that a greater skill mix in primary care may be supportive of self-management change and in enabling staff to further develop their skills. Patients with long-term conditions and/or who engage in health risk behaviours often lack motivation to seek help to change behaviour [55], so are less likely to pro-actively access services. And despite the evidence that supports the cost-effectiveness of behavioural/psychological interventions for long-term conditions, preventative health care and mild-moderate mental health conditions [56, 57], there are still significant known barriers to accessing these services. This is particularly so for ‘hard-to-reach’ groups [32, 33, 58]. Evidence also shows traditional behavioural interventions are less effective for some of these groups [59]. This indicates that a different and more targeted approach may be required. Integrated care of the kind we describe in this article could offer an approach that works to tackle these challenges.

## Evidence from other models of health care

When one looks beyond the UK context, it can be seen that integrated and collaborative models of primary care are not new. As described above, primary care services in the USA have been leading the way on integrated primary care, which involves professionals with behavioural/psychological expertise as part of a collaborative multi-disciplinary primary care team. Such integrated care is gaining momentum [60]. Organisations in the USA are now producing guides to integrated behavioural health care, and defining the different levels of integration that should be implemented. For example, Substance Abuse and Mental Health Services Administration – Health Resources and Service Administration (SAMHSA-HRSA) define a 6-level model of integrated care, specifying what needs to happen at each step to reach the specified level of integration [61]. Level 6 sees fully integrated and co-located teams functioning as one system, including behavioural and medical staff. It is driven by a shared concept of team care, involves formal and informal meetings to support integrated and collaborative practice, and the roles and cultures between staff groups blend and influence each other.

One such system that has successfully implemented level 6 integrated behavioural health care is the award-winning ‘Nuka’ system. This has been pioneered by the South Central Foundation (SCF), based in Alaska. Relationship-based continuity of care is central to the ethos of this system. Nuka has integrated primary healthcare teams who typically have a patient list of 1,200–1,400 patients. The team is co-located (when not seeing patients) and consists of a GP, a Case Manager (nurse), Health Care Assistant, administrator and BHC. The team manage patients’ care jointly; make team formulations, care plans and decisions in order to promote and maintain physical, mental, emotional and spiritual health and wellbeing. We suggest a similar approach to integrated behavioural health care within a primary care setting is required to address the current challenges in the UK context.

Nuka stands as an exemplar of integrated behavioural primary care. They have achieved remarkable improvements in health care indicators; for example, a 42 % reduction in accident and emergency visits and a reduction in strokes of 62 % over a 10 year period [62, 63]. It’s possible that such improvements may be more stark in the USA, given that typically a lower proportion of healthcare consultations are in the primary care setting than in the UK [64, 65]. Further, due to the varied models of funding, it is not standard to ‘assign’ each person with a primary care provider in the USA, like it is in the UK [65]. Therefore, the baseline with which to compare integrated care in the USA compared to the UK differ.

Other areas of the USA have successfully implemented similar integrated primary care systems, consisting of multidisciplinary teams addressing a broad range of problems [57, 66, 67]. This includes the Veterans Health Administration, which have also been leaders in the USA in delivering integrated care, incorporating improved access for patients, telemedicine, greater preventative medicine and resulting in improved outcomes, similar to that of SCF [68, 69]. Further, examples of effective models of integrated care can be seen in a range of higher-income countries, including Australia, Singapore and Germany, however not all have all facets of integrated care as defined in the USA [70–72]. The combination of integrated care teams, embedding behavioural expertise (a BHC) and adopting a collaborative, relationship-based ethos appears central to success. We endorse this view and believe that more exploration of this role in UK primary care is warranted.

In the USA models, the BHC supports the primary care team through on-going training, informal consultancy and daily interactions to improve collaborative, relationship-based, biopsychosocial working. This is done through contributing to joint care planning, advising staff on psychological/behavioural treatment and supporting/coaching staff to develop their own collaborative style with patients to promote behaviour change and strong relationships. BHCs also work directly with patients to improve management of long-term conditions, lifestyle behaviours and mental wellbeing. The role requires a post-graduate qualification (MSc level or higher) plus several years of post-qualification experience working in relevant areas (e.g. long-term conditions management and/or behaviour change in order to gain a license to practice). Therefore, BHCs have a background in psychological interventions and have expertise in delivering high-intensity behaviour change interventions to people with health problems, as well as working preventatively. Thus, a range of psychological/behavioural interventions to patients could be delivered depending on the needs of the patient, ranging from low to high intensity, and with a focus on medium-high intensity level interventions for complex cases that are amenable to relatively short-term psychological input.

## UK integration efforts and the role of the Behavioural Health Consultant

There are growing efforts to deliver care in integrated ways. Around the UK, attempts have been increasingly made to embed psychological/mental health expertise into primary care and other settings, bringing a greater level of integration [73–79]. These tend not to be fully integrated (i.e. level 6 as defined by SAMHSA-HRSA). For example, they may rely on referral systems for patients to receive psychological input, be located in the same building as medical staff but not the same office, and/or integrate case managers, but not have a fully integrated multi-disciplinary team. Archer and colleagues also note that collaborative care can vary enormously, warranting the use of definitions in trials to increase specificity and comparability [41].

Although many of the approaches utilised in the UK may represent a level of integration, they would be likely to relate to level 3 or 4 integrated care, which involves some direct collaboration and communication about patients’ needs, however this tends to be driven by patients labelled as ‘difficult’, and teams have typically have only a basic understanding of other members’ roles. Further, without the behavioural expertise aspect of an integrated care team, they would not - according to SAMHSA-HRSA - be defined as offering integrated behavioural health care [61]. Where there is a psychological role in UK trials, this tends not to have the breadth that the BHC role does; rather, it focuses only on mental ill health and/or long-term conditions, omitting any preventative element, and is therefore narrower in focus. Further, a systematic review of worldwide collaborative care interventions for depression and anxiety revealed that there were no secondary effects on physical health [41]. Yet, research shows significant mental health benefits of behaviour change programmes targeting patients’ lifestyle [80]. This suggests that behavioural expertise targeting physical health and lifestyle behaviours (e.g. diet, physical activity, alcohol use etc.), as well as depression, may be needed for broader outcomes in physical as well as mental health. The challenges of both delivering integrated care to patients with multiple morbidities and enabling staff from medical backgrounds to practice collaboratively in models of integrated care are acknowledged [74, 81, 82].

To our knowledge, the only UK primary care pilot to attempt to trial a level 6 integrated behavioural health care system in primary care was a ‘proof of concept’ pilot in a GP surgery in Fife, which both authors were involved with. This consisted of a GP, case manager, practice nurse, administrator and a BHC and was modelled on the integrated care system in SCF [83]. Therefore, all five members of staff were co-located when not seeing patients, which helped facilitate the coordination of care and learning from each other. The BHC coached staff to develop their skills in supporting patients with psychological difficulties, in managing long-term conditions, and using behaviour change approaches to help support lifestyle change. At times, patients who saw the GP for medications (for example sleeping pills) for problems that could be addressed behaviourally, would see the BHC in addition. Patients were always given a choice of which staff member to see and were encouraged to address difficulties behaviourally with support of the BHC, where appropriate. During the time period, Hannah and colleagues found that demand reduced, that there was a shift in balance to roughly half face-to-face and half telephone appointments, and that staff satisfaction improved [83]. Within this model, the BHC role aimed to work with staff and patients to support a collaborative care model, empowering patients to make changes to improve their health [84]. Integrating the BHC into the team may have broken down stigma that can be attached to behavioural/psychological care and enabled patients to be seen immediately, reaching hard-to-reach groups [84].

Fully integrated primary care models that are more established in the USA and other higher-income countries are relevant for UK primary care. The experience of the authors and Hannah and colleagues shows that this model helps a shift towards patient-centred care, collaborative-care and continuity of care. Therefore, fully integrated care perhaps goes beyond current thinking and policy around patient-centred care in the UK (For example, House of Care model, The Quality Strategy, the 2020 Vision) [85–87]. As a result, there is a need to consider fully integrated behavioural primary care systems for the UK context.

## What are the potential benefits of BHCs as part of an integrated primary care team?

The possible benefits of this approach for UK patients and staff within primary care services are numerous. In particular, the BHC role - as part of an integrated, collaborative primary care team - would likely enhance the real-world effectiveness of interventions [66]. This is, in part, through the greater reach of access to patients and delivery of early behavioural interventions, as well as through work with staff to integrate greater collaboration and behavioural/psychological approaches throughout the primary care team.

For patients, the integrated nature of BHCs within primary care teams will likely help ‘normalise’ behavioural/psychological treatment, with a consequent effect on reducing stigma, leading to increased patient trust. Some patients presenting in primary care will need to be referred on for longer-term psychological input. Benefits to wider psychological and behavioural services offering longer-term input around mental health and long-term conditions (for example, weight management in a diabetes population) would be seen through the BHC helping motivate patients for, and normalise input from such teams, better preparing patients for such services. This could have a beneficial impact on DNA rates and drop-out from programmes. The BHC also aids the maintenance of changes following input from longer term treatment, through liaising with mental health and long-term conditions services in secondary care.

The BHC would also support team members to develop skills in communication approaches such as motivational interviewing. This is complementary to, and further supports, shared decision making [88]. BHCs are specifically trained to bring a biopsychosocial formulation of patients’ presenting problems, supporting the whole team to implement this approach through shared care plans.

Further, since integrated team working fully values the contribution of each individual, it enhances communications and creates a better environment for staff and improved patient services [63]. These aspects will benefit staff in the collaborative team, helping them to feel empowered in providing care and supporting patients more effectively.

## What are the potential challenges of Behavioural Health Consultants as part of an integrated primary care team?

Challenges for patients may exist in taking a shared role in care and decision making. Patients are typically adapted to communicate with staff who operate a predominantly paternalistic model, yet have frustrations with the sometimes unrealistic goals a physician or nurse may suggest [89]. It could take time to support patients to play a greater role in their care (where this has not been the case previously). Some patients may resist moving to a new model of care, and staff may also need support from a BHC to help them achieve this [90]. Given that patients are allocated a single team, there could also be challenges if patients have a desire to see different doctors or nurses for different problems, for example seeing a staff member of a particular sex. These challenges are not insurmountable and could be addressed, for example, through allowing patients to see staff from a different team on occasions.

Challenges for staff and service commissioners include: the time taken to move towards collaborative and integrated care, resistance to changes in practice/ways of working, and the increased team working and supervision required for the staff team. Indeed, some team members may resist team working due to habit or preferred style, and therefore not provide a high level of integrated care within the team. Opportunity costs too are a key consideration here, since although greater investment of resources is needed, the benefits gained from the investment presumed to be greater than other options, as evidenced from the USA [62, 63, 91, 92]. Therefore, the evidence we present and the discussion of experience to date endorses the view that fully integrated care would be the best use of resources for maximum gain in population health. However, since existing staff (e.g. GPs and nurses) will spend a greater amount of time working within the team, than currently with patients, there may be challenges in gaining sufficient resource to enable to model to be successful.

Consequently, adapting to a new model of care delivery is extremely challenging. Pursuing systemic changes will be needed to meet the current and future needs of the population. It is clear from the USA and our recent experience that the BHC role is wider than purely providing psychological/behavioural interventions one-to-one with patients [62, 63, 83, 84]. This is one of the key strengths of this model and must be carefully considered as part of developing such a role within an integrated team.

## What changes are required to develop this approach in primary care in the UK?

To date within the UK, collaborative care models for psychological problems have tended to focus purely on management of anxiety and depression. Though trialed within RCTs and implemented in other countries, there is now an opportunity to embed a full behavioural/psychological approach in the UK, incorporating prevention and the management of long-term conditions in primary care through the model we suggest [41, 63].

There are clear differences between the health systems in the USA and the UK, not least in that in the latter, healthcare is free at the point of delivery. The intricacies of USA-style integrated primary care would therefore need to be carefully tailored to the local context – across the UK, regionally, and within specific practices. Involving community members in the development of services is likely to afford considerable benefit, helping to ensure that interventions are tailored to local needs and assist in overcoming common barriers to change. There may also need to be restructuring of physical space in GP surgeries to accommodate co-located integrated care teams (an essential part of integrated care).

Additionally, a shift in principles, values and attitudes is needed to enable the transformation of primary care, particularly in terms of moving towards a collaborative model that reduces hierarchical working and fully values the contribution of each team member (and the patient). This may be particularly challenging and some staff members in teams that seek to move towards a shared care model may struggle to adopt such a shift. Support for change needs to come from leaders, managers and staff working on the ground. Pump priming and involvement of organisational development may assist in ensuring the necessary staffing arrangements, and further support teams to work effectively together. This investment may be required for integrated care to work in practice, and for population-level changes in health to be achieved. In terms of economic costing, it is our belief that the increased investment in developing and sustaining integrated care will have greater long-term benefits than costs [91, 92].

There may also need to be wider strategic-level changes and changes to remuneration structures within primary care to support the broad adoption of this model. For example, the use of the Quality and Outcomes Framework (QOF) has been criticised as emphasising a biomedical and paternalistic model in primary care [93]. Although GP practices in the UK can opt-out of the QOF, most practices currently remain part of this reporting system. These systems need to move away from a focus on illness indicators and became more supportive of collaborative practice that works towards improving health and wellbeing. The QOF could, therefore, be further developed to enable remuneration for practices who adopt the principles and structures of integrated care, and for providing evidence-based interventions to support self management and lifestyle change. This could explicitly include indicators around offering behavioural health expertise to patients, as well as a focus on preventative activities. Doing so could encourage integrated ways of working.

There are relevant changes required for psychological/behavioural practitioners who wish to adopt the role of a BHC, within a fully integrated primary health team. The role is novel, complex and demanding, due to the breadth of areas targeted and the integration of behavioural health directly into a team, which enables opportunities to embed within the team, supporting improved communication and team-work. The BHC role is therefore emotionally demanding given the continual support given to GPs and other staff. Health (and other Applied) Psychologists are particularly well-placed to take on the BHC role but will require further training and supervision to adapt to shorter sessions, the breadth of work, and support colleagues to work more collaboratively/relationally [94].

## Summary and conclusions

This debate article has conveyed the authors’ view that a fully integrated model of behavioural/psychological expertise within a reimagined primary care team shows great promise for addressing the on-going pressures faced in UK general practice and psychological services. This approach would see a multi-disciplinary team being co-located and working collaboratively with each other and patients, which goes beyond current thinking in the UK around integrated care. We have argued for the benefits and opportunities associated with a relationship-based, empathetic and collaborative form of care that is centred around patients. It suggests the need for staff with a high level of behavioural expertise is required to support the role, which adds to broader discussions around integrated care. The benefits of this approach, based on evidence from the USA, and our own experience in the UK context, suggest resultant improvements for staff, patients and NHS services in a number of areas. These include: improved access to evidence-based interventions, particularly for hard-to-reach groups, enhanced real-world effectiveness, greater shared decision-making, improved prevention efforts, self-management of conditions and engaged staff with additional skills and expertise and reductions in healthcare utilisation.

Relevant guidelines for fully integrated models of care regard behavioural/psychological expertise as an integral part of the multi-disciplinary team. However, to date multi-disciplinary teams in primary care, and integrated care attempts in the UK have fallen short of the full integration that is required to attain the scale of improvements in outcomes required. One small-scale pilot of level 6 integrated primary care with BHCs in the UK demonstrated promising results but a wider trial over a longer period is needed to assess effectiveness of this approach. Larger scale piloting and trials are warranted, which should include full economic assessment. Although the most robust evidence can be gained from controlled trials, there can be limitations for a real world context, particularly due to standardisation and inclusion/exclusion criteria [95–98]. A combination of larger pilots (than those that have been undertaken in the UK to date) along with pragmatic randomised trials, which explore integrated care, and integrated care with BHCs (compared to usual care) is required to test this model. This may allow a balance to be struck in gaining robust trial data and practice-based evidence [99, 100]. Any trials or pilots may require some reorganisation of primary care structures, remuneration and outcome frameworks in order to deliver this model. Changes in staff attitudes and behaviours will be required as well, along with strong leadership to realise this approach, even in pilot or trial studies. A new role of BHC is required for such testing, and current behavioural/psychological practitioners (for example, Health/Applied Psychologists) will need further training and support to adapt their practice to take on the challenge of working effectively in this new primary care environment.

## Abbreviations

UK, United Kingdom; GP, general practitioner; IAPT, increasing access to psychological therapies; USA, United States of America; NHS, national health service; SCF, South Central Foundation; BHC, behavioural health consultant; QOF, quality and outcomes framework
